# Transcranial static magnetic field stimulation of the supplementary motor area decreases corticospinal excitability in the motor cortex: a pilot study

**DOI:** 10.1038/s41598-024-57030-0

**Published:** 2024-03-19

**Authors:** Cristina Pagge, Jaime Caballero-Insaurriaga, Antonio Oliviero, Guglielmo Foffani, Claudia Ammann

**Affiliations:** 1 HM CINAC (Centro Integral de Neurociencias Abarca Campal), Hospital Universitario HM Puerta del Sur, HM Hospitales, Madrid, Spain; 2 PhD Program in Neuroscience, Autonoma de Madrid University-Cajal Institute, Madrid, Spain 28029; 3 Escuela Técnica Superior de Ingenieros de Telecomunicación, Universidad Politécnica de Madrid, 28040 Madrid, Spain; 4 Champalimaud Research, Champalimaud Foundation, Lisbon, Portugal; 5 Hospital Nacional de Parapléjicos, SESCAM, Toledo, Spain; 6 CIBERNED, Instituto de Salud Carlos III, Madrid, Spain; 7 Faculty of Health Sciences - HM Hospitales, University Camilo José Cela, Villanueva de la Cañada, 28682 Madrid, Spain

**Keywords:** Neurophysiology, Diseases of the nervous system

## Abstract

Transcranial static magnetic field stimulation (tSMS) is a non-invasive brain stimulation technique that is portable and easy to use. Long-term, home-based treatments with tSMS of the supplementary motor area (SMA) are promising for movement disorders and other brain diseases. The aim of the present work was to investigate the potential of SMA-tSMS for reducing corticospinal excitability. We completed an open pilot study in which twenty right-handed healthy subjects (8 females; age: 31.3 ± 5.4 years) completed two 30-min sessions (at least one week apart) of SMA-tSMS. We assessed corticospinal excitability by applying transcranial magnetic stimulation (TMS) over the primary motor cortex, recording 30 motor evoked potentials (MEPs) from either the left or right first dorsal interosseous (FDI, ‘hotspot’ muscle) and extensor carpi radialis (ECR, ‘offspot’ muscle) in each session before and after (up to 30 min) tSMS. We observed moderate-to-extreme level of Bayesian evidence for a reduction of MEP amplitude after 30 min of tSMS over SMA compared to baseline. Thus, tSMS applied over SMA may reduce corticospinal excitability. These findings, if confirmed with double-blind, placebo-controlled experiments, support the potential of targeting the SMA for neuromodulating a large motor network in future therapeutic applications of tSMS.

## Introduction

Transcranial static magnetic stimulation (tSMS) is a non-invasive brain stimulation technique (NIBS) that consists in the application of a neodymium magnet to the scalp. Since the first study that proposed this method^1^, several others^2–8^ confirmed that tSMS can produce a decrease of corticospinal excitability when applied over the primary motor cortex (M1). In parallel, promising clinical applications that apply tSMS over M1 are emerging for amyotrophic lateral sclerosis^9^, Parkinson's disease^10^, and stroke^11^. Importantly, the portable and user-friendly nature of tSMS makes it appealing for home-based treatments.

Besides M1, another attractive target to stimulate with tSMS is the supplementary motor area (SMA). Due to its broad neuronal projections, the modulation of SMA could have an impact on the entire motor network resulting in a more effective stimulation, especially for the treatment of neurological and psychiatric disorders in which the control of movements or behaviors is altered, including Tourette syndrome^12–14^ obsessive compulsive disorder^15^, and Parkinson’s disease^16–18^. It has been already demonstrated that tSMS applied to the SMA induces both local changes in resting-state activity below the magnet and distant changes in connected cortical and subcortical motor regions, including the motor striatum and M1^19,20^. Early work with repetitive transcranial stimulation (rTMS) suggests that stimulating the SMA may modulate corticospinal excitability in M1^21^. The possibility that tSMS of the SMA may induce changes in corticospinal excitability in M1 is attractive for clinical applications, but remains unexplored.

To address this gap, we conducted an open pilot study to test the ability of tSMS applied over SMA for 30 min to produce after-effects on the excitability of M1. Our primary outcome was the change of motor evoked potentials (MEPs) measured with TMS. The secondary outcome was to evaluate the effects of tSMS applied over SMA on intracortical excitability.

## Experimental procedures

### Study design

This is an open pilot randomized study.

### Participants

We enrolled 20 (8 females; mean age: 31.3 ± 5.4 years) right-handed healthy volunteers. Exclusion criteria were: history of psychiatric and neurological disorders, traumatic brain injury, presence of metallic implants or implantable cardiac stimulators, consumption of medication that could affect the nervous system at the time of the study.

The study was performed according to the Declaration of Helsinki and approved by the local Ethics Committee at HM Hospitales, and participant signed informed written consent.

### Interventions

#### tSMS

**Figure 1:**
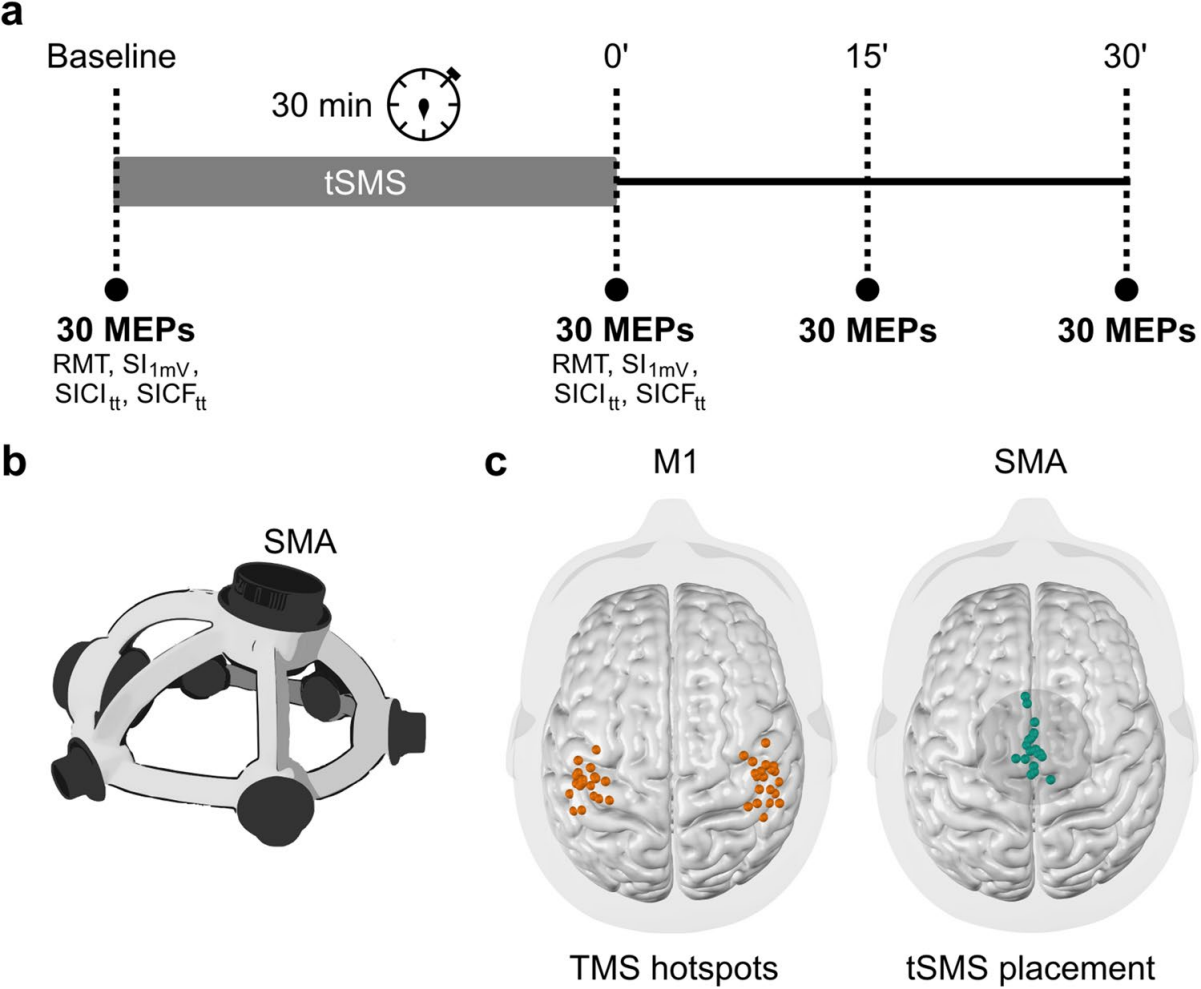
Experimental design (**a**) All measures (except MEP recording) were performed with threshold-tracking methods. (**b**) Helmet equipped with ø60 mm magnet (MAG60r^+^) for SMA stimulation. (**c**) Each subject's FDI hotspot location for both hemispheres (left) and each subject's location of the SMA-tSMS placement (right) obtained as the intersection of the TMS coil’s z-axis (using its position and orientation as extracted from BrainSight workstation in MNI space) with the convex hull of the outer brain surface (MNI152 nonlinear 2009c, asymmetric template). The magnet was placed in the MNI space just over the scalp, in the manually delineated medial line and 3 cm anterior to its midpoint (nasion to inion), oriented perpendicular to the scalp's surface. The base of the magnet is represented, and the viewpoint is centered at the axis of the magnet. The SMA-tSMS placement for one outlier was centered on the midline.

Each subject underwent two tSMS sessions for 30 min applied over SMA, at least one week apart (Fig. 1a). The participants were seated comfortably and were instructed to refrain from speaking and to remain awake while in a calm, relaxed state.

tSMS was delivered using a cylindrical neodymium magnet with N52 grade, 30 mm height and with a diameter of 60 mm (MAG60r + ; Neurek SL, Toledo, Spain). It was applied with south polarity over the SMA, centered 3 cm anterior to Cz (defined accordingly to the international 10–20 system for EEG), using a specifically designed helmet (Fig. 1b) to target this cortical area (MAGsv1.1; Neurek SL, Toledo, Spain). The overall weight of the helmet was sustained with a flex arm equipped with a spring of equivalent force.

### Outcomes

Our primary outcome was the tSMS-induced modulation of M1 corticospinal excitability as measured by MEP amplitude elicited with TMS.

We used a 70-mm figure-of-eight-shaped magnetic coil to perform monophasic TMS through a Magstim BiStim^2^. The coil was held tangential to the scalp with the handle oriented backwards and 45° from the midline. The induced current presented a posterior-anterior (PA) direction activating preferentially I1 waves^22,23^. Intensities were expressed as a percentage of the maximum stimulator output (%MSO). To ensure the stability of the coil position, we used a frameless neuronavigation system (BrainSight; Rogue Research).

TMS was always delivered over the M1 area that corresponded to the first dorsal interosseous (FDI) hotspot. Additionally, extensor carpi radialis (ECR) recordings were used to investigate the effect of SMA-tSMS in an ‘offspot’ muscle not optimally targeted by TMS. The resting motor threshold (RMT) of each individual was defined as the %MSO required to elicit a MEP in the FDI muscle of at least 0.05 mV using a threshold-tracking method based on the maximum-likelihood Parameter Estimation by Sequential Testing (PEST) strategy without a priori information (freeware program of TMS Motor Threshold Assessment Tool, MTAT 2.0^24^). Each threshold assessed with this method was obtained delivering typically 12 magnetic pulses, unless the tracking did not converge. In this case (which is rare in healthy subjects) the number of pulses was increased until reaching the optimal tracking (max. 30). For each measurement we needed approximately 2 min. The stimulus intensity used to record the MEPs was determined with the same method, with a target MEP peak-to-peak amplitude of 1 mV.

At baseline, we recorded 30 MEPs either of the right or the left hemisphere. Post-tSMS measurements consisted of 30 MEPs recorded with the same intensity immediately after (P_0_), 15 min (P_15_) and 30 min (P_30_) after tSMS application.

Our secondary outcome was to test whether 30 min of tSMS over SMA can produce changes in intracortical excitability.

To evaluate intracortical excitability we, again, employed the threshold-tracking method to the FDI hotspot. The intensity of the control test stimulus (TS) was defined as the stimulus intensity required to elicit a target MEP of 0.2 mV. For both short-interval intracortical inhibition (SICI) and short-interval intracortical facilitation (SICF), the conditioning stimulus (CS) intensity was set at 80% of the RMT. In the SICI protocol the CS preceded the TS by 2.5 ms in order to reach maximum inhibition^25^ and for SICF the CS appeared 1.4 ms after the TS^6^. The intensity of the TS was adjusted to ensure that the target MEP amplitude of 0.2 mV was maintained in the presence of the CS. We continued adjusting the intensity until obtaining the best estimate of the optimal intensity for eliciting the target response. The threshold-tracking measurements for TS intensity (0.2 mV), conditioned TS intensity for SICI and SICF were repeated after the 30 MEPs at P_0_. The inter-trial interval for all TMS protocols was 6 s ± 10%. The complete experimental protocol had approximately a total duration of one and half hour and is represented in Fig. 1a.

As an exploratory (ancillary) analysis we computed the distance between the FDI hotspot and the SMA location of each subject. The experimental procedure involved the use of a frameless neuronavigation system (BrainSight; Rogue Research) to precisely target specific brain regions during TMS. The subject's head and TMS coil were simultaneously tracked by an infrared camera using sensors placed over the coil and the subject's head. A template MNI scan provided by the software was registered into subject space by identifying the subject's nasion and right and left tragi. The FDI hotspot and SMA location were established and saved in the system. Throughout TMS, the coil deviation from the target was monitored in real-time through a bull's eye displayed on screen, which provided information about the distance to the target and the angular error.

### Sample size

We aimed for 20 participants since the Bayesian evidential strength to provide evidence for or against an effect is reasonable starting from this sample size^26^.

### Randomization

The neurophysiological assessment with TMS was performed over the right hemisphere in one session, and over the left hemisphere in the other session. The order of hemispheres was randomized using coin flip.

## Analytical methods

### Data analysis

To estimate single-trial MEP amplitude, we measured the EMG response within a 10–60 ms window after the TS. Mean MEP amplitudes were calculated by averaging the MEP values across subjects for each time point for the FDI and ECR, and these were reported as mean values ± SD. We also calculated normalized MEP amplitudes as 100 × [(MEPpost-MEPpre)/MEPpre]. SICI and SICF were calculated offline as percentage change based on the equation: –100 × (conditioned TS intensity – non-conditioned TS intensity)/non-conditioned TS intensity, so that negative values represent inhibition and positive values facilitation.

The positions of all FDI hotspots and SMA tSMS locations were obtained offline by calculating the intersection of the TMS coil’s z-axes (using its position and orientation extracted from the BrainSight workstation in MNI space) with the convex hull of the outer brain surface (MNI152 nonlinear 2009c, asymmetric template). The distance between the FDI hotspots and SMA was computed as a Euclidean distance between the two cortical locations, centering the SMA location over the midline to reduce medio-lateral variability.

### Statistical analyses

We used Bayesian statistics with default effect size priors (Cauchy scale 0.707) to perform all statistical analyses in JASP (version 0.17). MEP amplitudes were analysed using two-way ANOVA, with TIME (Baseline, P_0_, P_15_, P_30_) as repeated measure factor and HEMISPHERE (left, right) as independent measure factor. We reported the BF_incl_ for the inclusion of a particular effect, which is calculated as the ratio between the likelihood of the data given the model vs. the next simpler model without that effect, comparing across matched models. Post-hoc comparisons were performed with *t*-tests, assuming directionality of change (post measures < baseline measures). Frequentist Bonferroni-corrected two-tailed *t*-tests (i.e. significance at *p* < 0.05/3 = 0.0167) were also reported for completeness. Comparisons of normalized (post/pre) values were performed with one-sample *t*-tests. Paired *t*-test results and correlations were reported using either one-tailed Bayes factors (BF_+0_ or BF_-0_) or two-tailed Bayes factors (BF_10_), depending on whether the directionality of the tested effect was expected a priori or not. Note that one-tailed Bayes factors are recommended in Bayesian statistics because they provide a fairer balance between the ability to provide evidence for the null hypothesis H_0_ or the alternative hypothesis H_1_^26^. We reported effect size estimates as median posterior Cohen’s *d* with 95% credibility intervals. Evidence in favour to alternative hypothesis (*BF* > *1*) or to the null hypothesis (*BF* < *1*) was described according to standard levels: anecdotal (*1/3* < *BF* < *3*), moderate *(*< *1/3 or* > *3*), strong *(*< *1/10 or* > *10*), very strong (< *1/30 or* > *30*), extreme (< *1/100 or* > *100*). Box plot horizontal lines represent median (Q2), first quartile (Q1) and third quartile (Q3); whiskers: minimum and maximum value excluding outliers.

## Results

### Participants flow

All 20 recruited subjects completed the study.

### Recruitment

Subjects were recruited between December 2019 and January 2021.

### Baseline data

The main demographics and neurophysiological characteristic are represented in Table 1. The localizations of the hotspots used and the SMA tSMS location for each subject are illustrated in Fig. 1c. Detailed statistical results and effect sizes are presented Table 2.

**Table 1:** Demographics and neurophysiological characteristics.

Sample size	20
Age (years)	31.3 ± 5.4
Gender (Females:Males)	8:12
RMT (%MSO)	45.2 ± 6.7
MEP amplitude (mV)	1.43 ± 0.60

Values reported as mean values ± SD.

**Table 2:** Detailed statistical results.

	Value	SD	% change	*p-value*	BF	δ	95% C.I
RMT (%MSO) (Baseline)	45.2	6.7					
RMT (%MSO) (P_0_)	45.1	7.5	− 0.2	0.775	0.14	0.04	− 0.26; 0.34
1 mV (%MSO) (Baseline)	55.6	12.9					
1 mV (%MSO) (P_0_)	56.6	13.2	1.8	0.246	0.57	− 0.17	− 0.48; 0.13
FDI MEP amplitude (mV) (Baseline)	1.43	0.60					
FDI MEP amplitude (mV) (P_0_)	1.14	0.59	− 20.28	0.001	49.6	− 0.52	− 0.85; − 0.19
FDI MEP amplitude (mV) (P_15_)	1.21	0.62	− 15.38	0.007	10.6	− 0.42	− 0.74; − 0.10
FDI MEP amplitude (mV) (P_30_) (n = 39)*	1.19	0.58	− 16.78	0.027	3.6	− 0.34	− 0.66; − 0.03
FDI MEP amplitude (norm P_0_)	0.82	0.37	− 18.00	0.004	17.1	− 0.45	− 0.77; − 0.13
FDI MEP amplitude (norm P_15_)	0.85	0.32	− 15.00	0.007	11.3	− 0.42	− 0.74; − 0.11
FDI MEP amplitude (norm P_30_) (n = 39)*	0.90	0.41	− 10.00	0.145	0.88	− 0.22	− 0.53; 0.09
FDI MEP amplitude (norm POST TOT)	0.86	0.31	− 15.00	0.006	14.2	− 0.43	− 0.76; − 0.12
ECR MEP amplitude (mV) (Baseline)	0.99	0.59					
ECR MEP amplitude (mV) (P_0_) (n = 39)*	0.77	0.43	− 22.22	< .001	439.1	− 0.65	− 1.00; − 0.31
ECR MEP amplitude (mV) (P_15_) (n = 39)*	0.88	0.53	− 11.11	0.004	16.6	− 0.45	− 0.78; − 0.13
ECR MEP amplitude (mV) (P_30_) (n = 37)**	0.82	0.48	− 17.17	< .001	67.95	− 0.56	− 0.91; − 0.22
ECR MEP amplitude (norm P_0_) (n = 39)*	0.81	0.32	− 19.00	< .001	90.7	− 0.56	− 0.90; − 0.23
ECR MEP amplitude (norm P_15_) (n = 39)*	0.90	0.30	− 10.00	0.044	2.3	− 0.31	− 0.63; 0.00
ECR MEP amplitude (norm P_30_) (n = 37)**	0.86	0.37	− 14.00	0.029	3.4	− 0.35	− 0.67; − 0.03
ECR MEP amplitude (norm POST TOT)	0.86	0.28	− 14.00	0.003	27.0	− 0.48	− 0.82; − 0.16
SICI_tt_ (%) (Baseline)	− 25.6	25.6					
SICI_tt_ (%) (P_0_)	− 23.2	25.9	− 9.4	0.535	0.21	0.09	− 0.21; 0.39
SICF_tt_ (%) (Baseline)	13.8	5.2					
SICF_tt_ (%) (P_0_)	13.8	6.7	0.0	0.968	0.17	− 0.01	− 0.30; 0.29

*p*-values and Bayes Factor (BF) results for paired *t*-tests from comparisons against the corresponding baseline or one-sample *t*-tests for normalized values, considering hemispheres as independent samples. Bonferroni-corrected significance is at *p* < 0.0167.

FDI, First dorsal interosseous; ECR, Extensor carpis radialis; MSO, Maximum stimulator output; C.I., Credible interval; norm, normalized, POST TOT, Grand-average of the post measurements.

*n = 39 (due to missing data).

**n = 37 (due to missing data).

### Numbers analysed

All participants were included in the analysis. Missing data were due to possible damage to EMG electrodes, which affected the complete ECR recording of one subject, P_30_ for ECR of a second subject, and P_30_ for ECR and FDI of a third subject.

### Outcomes and estimation

#### tSMS over SMA can modulate corticospinal excitability measured from M1

The primary outcome of this study was to investigate whether 30 min of tSMS over SMA can modulate corticospinal excitability.

**Figure 2:**
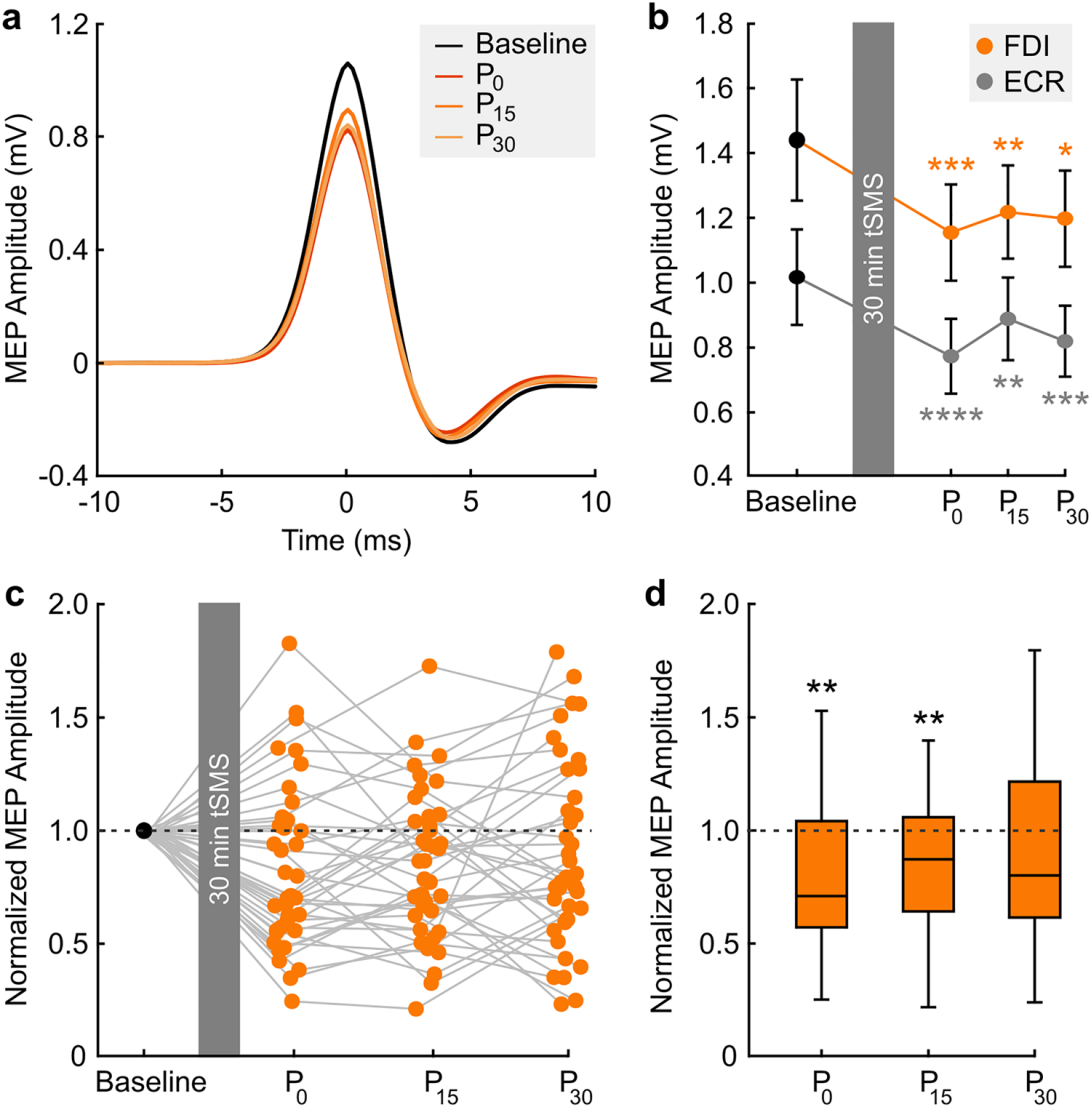
Effects of 30-min tSMS over SMA on corticospinal excitability. (**a**) Grand-average of FDI MEP amplitudes obtained by averaging all trials after temporal re-alignment of each trial to the MEP peak. Data shown for baseline (Base) and post-tSMS measurements (P_0_, P_15_, P_30_). (**b**) Effects of 30-min tSMS on raw MEP amplitudes of FDI and ECR muscle. Repeated measures ANOVA: FDI—TIME, F_3,111_ = 4.6; BF_incl_ = 8.3; ECR—TIME, F_3,105_ = 9.6; BF_incl_ = 1479.5. Error bars represent the 95% credible interval. (**c**) Individual MEP amplitudes of FDI for each post-tSMS time point normalized to baseline. (**d**) Mean MEP amplitudes of FDI for each post-tSMS time point normalized to baseline. One-sample *t*-tests. *BF > 3 (moderate); **BF > 10 (strong); ***BF > 30 (very strong); ****BF > 100 (extreme).

We obtained moderate level of evidence for a reduction of MEP amplitude (primary outcome) after 30 min of tSMS over SMA for the FDI (two-way ANOVA, TIME, F_3,111_ = 4.6; BF_incl_ = 8.3; Fig. 2a,b). The effect was independent of the hemisphere (TIME*HEMISPHERE, F_3,111_ = 0.54; BF_incl_ = 0.13). The decrease in MEP amplitude was maintained for at least 30 min after the end of the stimulation (Fig. 2a,b) and was observed at all three time points compared to baseline with moderate to very strong evidence (*t-*test, P_0_: BF_-0_ = 49.6; P_15_: BF_-0_ = 10.6; P_30_: BF_-0_ = 3.6). Similar results were found when MEP amplitudes were normalized to baseline (Fig. 2c,d). With respect to the ECR we found extreme evidence for the reduction of the MEP amplitude compared to baseline (TIME, F_3,105_ = 9.6; BF_incl_ = 1479.5; Fig. 2b) and it was consistently observed at all 3 time points with strong to extreme evidence (*t-*test, P_0_: BF_-0_ = 439.1; P_15_: BF_-0_ = 16.6; P_30_: BF_-0_ = 67.9). Again, the effect was independent of the hemisphere (TIME*HEMISPHERE, F_3,105_ = 1.94; BF_incl_ = 0.59). When adding gender as a factor in the ANOVAs, we found moderate evidence supporting that gender had no impact on the results both in FDI (TIME*GENDER, F_3,105_ = 0.91; BF_incl_ = 0.19) and ECR (TIME*GENDER, F_3,99_ = 0.33; BF_incl_ = 0.12). Additionally, we found moderate evidence of absence for an increase of RMT after tSMS compared to baseline (BF_+0_ = 0.14) and anecdotal evidence of absence (BF_+0_ = 0.57) for a raise in 1 mV-intensity. For the values of effect size see Table 2.

This first result shows that tSMS applied over SMA can reduce corticospinal excitability for at least 30 min after the intervention.

#### tSMS over SMA does not modulate intracortical excitability

As our secondary outcome, we evaluated whether the induced tSMS-effects were associated with changes of intracortical excitability, assessing SICI and SICF before and after the application of 30 min of tSMS over SMA.

We found moderate evidence of absence for changes in SICI (Baseline: -25.6% ± 25.6%; P_0_: -23.2% ± 25.9%; BF_10_ = 0.21) and in SICF (Baseline: 13.8% ± 5.2%; P_0_: 13.8% ± 6.7%; BF_10_ = 0.17) compared to baseline, both calculated over the FDI response. For the values of effect size see Table 2. Therefore, intracortical excitability was not modulated by 30-min tSMS over SMA.

### Ancillary analysis

The distance between the SMA target and the FDI hotspot in standard space was 54.7 ± 9.5 mm and did not correlate with the tSMS-induced decrease in MEP amplitude expressed as percent change from baseline either of FDI MEPs at P_0_ (Pearson's r = − 0.10, BF_+0_ = 0.14), or grand-average FDI MEPs at [P_0_,P_15_,P_30_] (r = − 0.36, BF_+0_ = 0.07), or grand-average FDI and ECR MEPs at [P_0_,P_15_,P_30_] (r = − 0.32, BF_+0_ = 0.07).

### Harms

None of the subject reported adverse events.

## Discussion

The present study shows that tSMS applied over the SMA can reduce corticospinal excitability measured in M1 for at least 30 min after the end of stimulation. This pilot finding supports the potential of SMA-tSMS as a neuromodulatory intervention to target the entire motor network.

The application of tSMS over the SMA reduced MEP amplitude in M1, without modulating SICI or SICF. This scenario is consistent with earlier findings (also in an open design) showing that 5 Hz repetitive TMS (rTMS) over the SMA of healthy individuals increased MEP amplitude in M1 with no changes in intracortical excitability^21^. Interestingly, the use of rTMS to neuromodulate the SMA did not allow to disentangle whether the changes in M1 corticospinal excitability were due to local changes in the SMA tonic activity ultimately affecting M1 through cortico-cortical projections (i.e. inducing plasticity both in SMA and in M1), or to the direct activation of SMA-M1 cortico-cortical projections without affecting local SMA activity (i.e. inducing plasticity in M1 but not in SMA)^21^. In this regard and in opposition to TMS, tSMS is unlikely to produce neuronal activation, providing insight into disentangling the mechanisms involved in the reduction of M1 excitability when tSMS is applied to SMA. In particular, it suggests that the reduction of corticospinal excitability we observed in M1 was probably mediated by tSMS-induced local plasticity in SMA, with a modulation of intrinsic SMA activity that, propagating through cortico-cortical projections, ultimately modulated corticospinal excitability in M1. This interpretation is supported by recent investigations showing a tSMS-induced modulation of SMA activity that produced changes beyond the local circuits^19,20^. Overall, tSMS of the SMA modulates local SMA activity^19,20^, cortico-cortical functional connectivity with connected areas^19^, corticostriatal activity^20^, and corticospinal excitability in M1.

Indeed, we cannot exclude that SMA-tSMS, with the relatively large magnet employed here (diameter 6 cm), modulated also the activity of close-by motor regions, particularly the most medial part of the dorsal premotor cortex, and/or part of the leg representation of M1. Modulation of these regions might have contributed to the overall reduction of corticospinal excitability we observed in the upper limb representation of M1. However, the possibility of a direct modulation of the hand representation of M1 through SMA-tSMS seems unlikely, since the distance between the SMA target and the FDI hotspot resulted to be relatively large and did not correlate with the reduction of MEP amplitude. In any case, the reduction in corticospinal excitability was robust regardless of the targeted hemisphere or whether the measurement was obtained from the hotspot muscle (the FDI) or an 'offspot' muscle (the ECR). These findings suggest a relatively broad bilateral neuromodulation induced by tSMS of the SMA.

Here we obtained a reduction of MEP amplitude of approximately 20% measured at the end of the tSMS application. This amount of reduction of corticospinal excitability induced by SMA-tSMS is comparable with the findings of previous investigations that applied tSMS for up to 30 min directly over M1 reporting consistently a decrease of 20–30% in MEP amplitude^1–4,6,8^. This inhibitory modulation of SMA opens new possibilities for therapeutic applications, especially for neurological and psychiatric disorders that compromise the control of movements. In line with this, a few investigations already described promising findings using M1-tSMS in patients with amyotrophic lateral sclerosis^9^, levodopa-induced dyskinesias^10^, and stroke^11^. While the therapeutic potential of SMA-tSMS has yet to be explored, other inhibitory non-invasive neuromodulatory techniques such as repetitive TMS or transcranial direct current stimulation applied to SMA have already shown beneficial effects in different pathologies especially when administered for multiple sessions^17,27,28^. In this context, long-term treatments with tSMS could be conveniently performed in a home-based manner by the patient, since the technique is portable and easy to apply. First investigations already exploited this advantage of tSMS performing multiple home-based sessions in patients with Parkinson's disease^10^.

It is important to acknowledge the main limitation of the present study. This was a pilot study, so all stimulation sessions used real tSMS. Consequently, a sham-controlled, randomized blind study is needed to confirm our findings.

In conclusion, our pilot study suggests that tSMS of the SMA may decrease corticospinal excitability in M1, which should be confirmed in future double-blind sham-controlled studies. Overall, tSMS applied to SMA seems an appealing protocol for clinical applications especially when a diffuse and bilateral neuromodulation of motor networks is preferred.

### Supplementary Information

Supplementary Information.

## Data Availability

The data that support the findings of this study are available as Supplementary Material.

## Abbreviations

tSMS: Transcranial static magnetic field stimulation
NIBS: Non-invasive brain stimulation
M1: Primary motor cortex
SMA: Supplementary motor area
MEP: Motor evoked potential
FDI: First dorsal interosseous
ECR: Extensor carpi radialis
SICI: Short-interval intracortical inhibition
SICF: Short-interval intracortical facilitation
BF: Bayes factor
ANOVA: Analysis of variance
rTMS: Repetitive transcranial magnetic stimulation
